# Expression and specificity of a chitin deacetylase from the nematophagous fungus *Pochonia chlamydosporia* potentially involved in pathogenicity

**DOI:** 10.1038/s41598-018-19902-0

**Published:** 2018-02-01

**Authors:** Almudena Aranda-Martinez, Laia Grifoll-Romero, Hugo Aragunde, Enea Sancho-Vaello, Xevi Biarnés, Luis Vicente Lopez-Llorca, Antoni Planas

**Affiliations:** 1 Laboratory of Plant Pathology, Department of Marine Sciences and Applied Biology, Multidisciplinary Institute for Environmental Studies Ramón Margalef, University of Alicante, PO box 99, 03080 Alicante, Spain; 2 Laboratory of Biochemistry, Institut Químic de Sarrià, Universitat Ramon Llull, Via Augusta 390, 08017 Barcelona, Spain

## Abstract

Chitin deacetylases (CDAs) act on chitin polymers and low molecular weight oligomers producing chitosans and chitosan oligosaccharides. Structurally-defined, partially deacetylated chitooligosaccharides produced by enzymatic methods are of current interest as bioactive molecules for a variety of applications. Among *Pochonia chlamydosporia* (*Pc*) annotated CDAs, gene *pc_2566* was predicted to encode for an extracellular CE4 deacetylase with two CBM18 chitin binding modules. Chitosan formation during nematode egg infection by this nematophagous fungus suggests a role for their CDAs in pathogenicity. The *P. chlamydosporia* CDA catalytic domain (*Pc*CDA) was expressed in *E. coli* BL21, recovered from inclusion bodies, and purified by affinity chromatography. It displays deacetylase activity on chitooligosaccharides with a degree of polymerization (DP) larger than 3, generating mono- and di-deacetylated products with a pattern different from those of closely related fungal CDAs. This is the first report of a CDA from a nematophagous fungus. On a DP5 substrate, *Pc*CDA gave a single mono-deacetylated product in the penultimate position from the non-reducing end (ADAAA) which was then transformed into a di-deacetylated product (ADDAA). This novel deacetylation pattern expands our toolbox of specific CDAs for biotechnological applications, and will provide further insights into the determinants of substrate specificity in this family of enzymes.

## Introduction

*Pochonia chlamydosporia* (Goddard) Zare and Gams is a nematophagous fungus which infects females and eggs of cyst or root-knot nematodes (RKN)^1–3^. It is a biocontrol agent against a number of plant parasitic nematodes in food-security crops such as tomato and barley^4^. *P. chlamydosporia* is also a soil saprophyte and a root endophyte^5–7^. Extracellular enzymes have been related with nematode egg infection^8^. Chitinases and especially proteases^7,9,10^ are considered potential virulence factors for degradation of egg-shell components. Interestingly, the recently sequenced *P. chlamydosporia* genome^11^ revealed a number of differentially expressed genes encoding for chitin modifying enzymes during the nematode infection process^12^.

Chitin, a linear polysaccharide of β-1,4-linked N-acetylglucosamine residues, is widely distributed in nature, being the major structural component of the exoskeletons of arthropods (including insects and crustaceans) and the fungal cell wall. Chitin is also present in the endoskeletons of mollusks, and in the cell wall of diatoms^13,14^. Chitin is depolymerized by chitinases, and deacetylated by the action of chitin deacetylases (CDAs) leading to chitosans and chitooligosaccharides, characterized by their degree of polymerization (DP), degree of acetylation (DA), and pattern of acetylation (PA). Chitin deacetylases (EC 3.5.1.41) belong to family 4 of carbohydrate esterases (CE4 in the Carbohydrate Active Enzyme classification, www.cazy.org)^15^ together with rhizobial NodB chitooligosaccharide deacetylases (EC 3.5.1.-), peptidoglycan N-acetylglucosamine deacetylases (EC 3.5.1.104), peptidoglycan N-acetylmuramic acid deacetylases (EC 3.5.1.-), acetyl xylan esterases (EC 3.1.1.72), and poly-β-1,6-N-acetylglucosamine deacetylases (EC 3.5.1.-). All CE4 enzymes share the NodB homologous domain^16^, with a distorted (β/α)_8_ barrel structure^17^ that contains the catalytic active site.

Chitin deacetylases (CDAs) play diverse biological functions. In bacteria, CDAs are involved in the catabolism of chitin (*i.e*. marine bacteria of the *Vibrionaceae* family for nitrogen recycling in chitinous debris^18^) or in signalling events (*i.e*. Rhizobia CDAs for Nod factors biosynthesis^19,20^). In fungi, they participate in cell wall morphogenesis and integrity, spore formation, germling adhesion, and fungal autolysis^17,21–25^. Fungal plant pathogens secrete CDAs during infection and early growth phase in the host to evade the plant defense mechanisms triggered by plant chitinases^17^. It has been hypothesized that partial deacetylation of their cell wall chitin or of the chitooligosaccharides (COS) produced by chitinases results in partially deacetylated oligomers that, unlike chitin oligosaccharides, are not well recognized by plant receptors reducing elicitation of plant defenses^26^. Few CDAs have been biochemically characterised with regard to substrate specificity: bacterial CDA such as *Rhizobium meliloti*^27^ or *Vibrio cholerae*^28,29^ and fungal CDAs such as *Mucor rouxii*^30^, *Aspergillus nidulans*^31,32^, *Colletotrichum lindemuthianum*^33,34^, *Puccinia graminis*^35^, *Pestalotiopsis* sp.^26^, and *Podospora anserina*^36^. They show different specificities on chitooligosaccharides leading to chitosan oligosaccharides with different patterns of acetylation as the result of random, sequential or processive mechanisms^17^. This rises the question on the role of the deacetylation pattern in the biological functions of CDAs.

Chitin is also a structural component of the eggshell of RKN which is the main barrier to pathogens^37^ including the nematophagous fungus *P. chlamydosporia*. The fungus genome contains three putative CDA-encoding genes^11,12^ of unknown function. Previous results detected chitosan formation in nematode eggs infected by *P. chlamydosporia*. Chitosan is associated with the sites of fungal penetration^12^, suggesting that *P. chlamydosporia* CDAs are involved in nematode infection. However, no studies have been carried out on the activity of these predicted CDAs.

In this work, we report the analysis of *P. chlamydosporia pc_2566-*encoded putative CDA protein (named *Pc*CDA hereafter) in order to unravel structure-function relationships with regard to specificity and pattern of deacetylation. In a broader context, novel CDAs need to be characterized to: a) decipher the determinants of specificity leading to chitosan oligomers with different acetylation patterns, and b) enlarge the toolbox of CDAs to generate well-defined chitosan oligosaccharides with biological activities. After expressing the *Pc*CDA catalytic domain in recombinant *E.coli*, the enzyme was purified from inclusion bodies by refolding and affinity chromatography. We demonstrate that the enzyme is indeed a deacetylase active on chitooligosaccharides with a novel deacetylation pattern compared to currently known CDAs.

## Results

### *Pc_2566* gene and encoded protein sequence

Genome sequencing and annotation predicted gene *pc_2566* (1368 bp ORF) as a putative chitin deacetylase^11^ (*Supplementary Information*, Figure S1A). Augustus and GeneMark gene predictors were coincident in the first two introns but not conclusive on other potential introns at the 3′-end (Figure S2). Sequence alignment with the highly homologous (70% identity) *Metarhizium acridum* NW_006916702.1 nucleotide sequence matched the first two introns in the sequences of both phylogenetically close fungi and identified the position of a third intron (Figure S3). The *Pc*CDA translated protein sequence contains a N-terminal signal peptide from residues 1 to 18 (Figure S1). The mature protein (after signal peptide removal) is composed of 455 amino acids with a calculated molecular mass of 48.7 kDa, an isoelectric point of 7.7, and exhibits one potential N-linked glycosylation site and six potential O-glycosylation sites. The catalytic domain (CE4 domain, residues 107 to 303) is flanked by two (N- and C-terminal) CBM18 modules (residues 30 to 74 and 360 to 441, respectively). These family 18 carbohydrate binding modules are typically involved in chitin binding^38^. *Pc*CDA full-length protein includes 25 cysteine residues, of which only two are located in the CE4 catalytic domain.

### Cloning, expression and purification of *Pc*CDA catalytic domain

**Figure 1:**
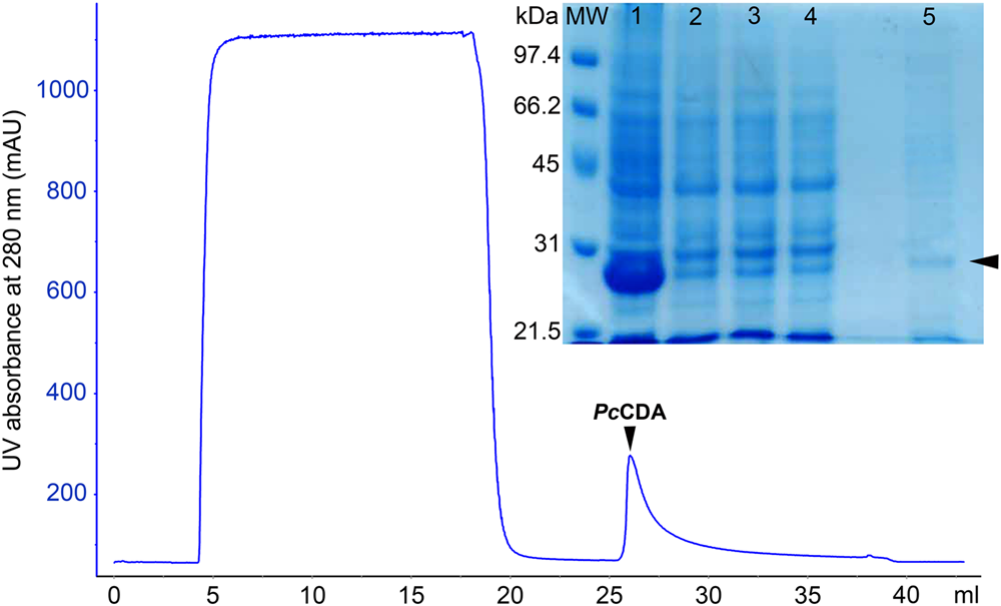
Affinity chromatography purification of *Pc*CDA catalytic domain. (**A**) Elution profile monitored by absorbance at 280 nm. (**B**) SDS PAGE analysis of fractions: Lane 1, sample after refolding by dialysis. Lanes 2 and 3, sample after centrifugation and filtered (0.45 μm) loaded into the column. Lane 4, flow-through from the column. Lane 5, eluted fraction with 2.5 mM d-Desthiobiotin after concentration by ultrafiltration. Arrow indicates column elution using d-Desthiobiotin. Arrowhead points to bands with the expected size (26.8 kDa) for *Pc*CDA catalytic domain.

The codon-optimized nucleotide sequence of the CE4 domain was subcloned into a pET22b vector for expression in *E. coli* (Figure S4). The expressed protein has a C-terminal Strep tag for purification by affinity chromatography (Figure S1B) and a predicted molecular mass of 26.8 kDa. All expression attempts varying temperature and time of induction rendered high protein expression but in the insoluble fraction after cell lysis. Solubilisation and refolding steps were necessary to obtain soluble and active protein. Inclusion bodies were solubilized in 7 M urea and soluble protein was recovered after refolding by dialysis (Figure S5) and purified by Strep tag affinity chromatography (Fig. 1). The eluted protein had an apparent molecular mass of 26.8 kDa in agreement with the expected mass. MALDI-TOF-MS analysis after in-gel trypsin digestion confirmed the identity of the *Pc*CDA catalytic domain. The overall yield was low (0.43 mg per L of culture) but sufficient for enzyme characterization.

### Deacetylase activity and specificity on chitooligosaccharides

**Figure 2:**
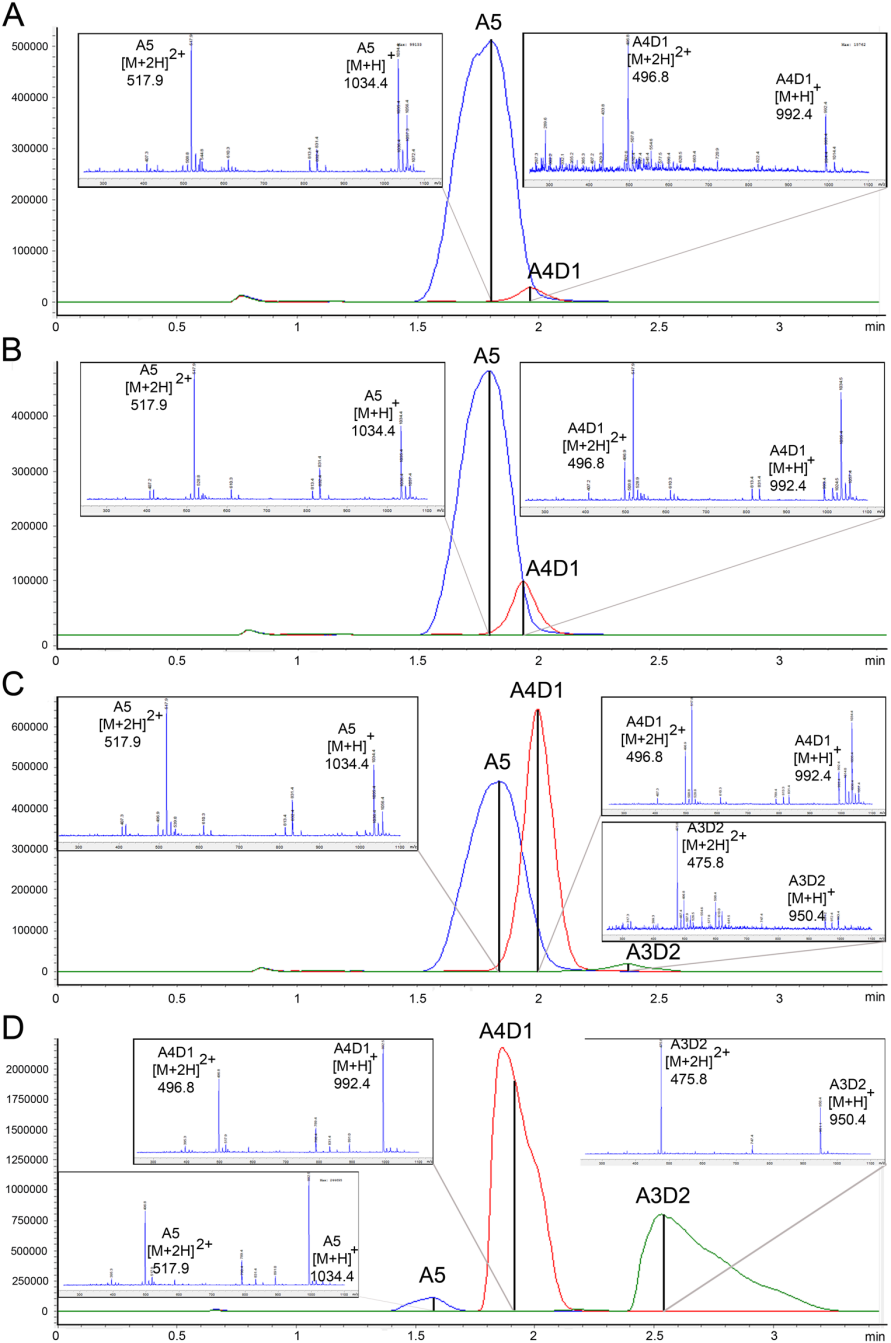
HPLC-MS monitoring of deacetylase activity by *Pc*CDA catalytic domain on GlcNAc_5_. substrate. Chromatograms show the presence of the substrate (A5) and the formation of mono-deacetylated (A4D1) and di-deacetylated (A3D2) products at different reaction times: (**A**) 10 min, (**B**) 5 h, (**C**) 24 h, and (**D**) 100 h. Reaction conditions: 0.2 mM substrate, 3.2 nM enzyme, 50 mM K_2_HPO_4_, 300 mM NaCl, pH 8.0, 37 °C.

*Pc*CDA catalytic domain was assayed for deacetylase activity on GlcNAc_3_ (A3) GlcNAc_4_ (A4), and GlcNAc_5_ (A5) substrates. Reaction mixtures were analysed by HPLC-MS at different incubation times to monitor products formation. Using the A5 substrate, *Pc*CDA generated a mono-deacetylated product after 5 h of reaction (Fig. 2). A di-deacetylated product appeared after 24 h. A5 was almost completely consumed after 100 h reaction, when both mono-deacetylated and di-deacetylated products were present and no further deacetylation was observed. With the A4 substrate, the enzyme was also active but slower than with the A5 substrate. A mono-deacetylated product was formed after 16 h of reaction and a di-deacetylated product appeared only after 100 h reaction while A4 was still present in a significant amount (Figure S6). *Pc*CDA had no activity on the A3 substrate (Figure S7), indicating that *Pc*CDA is active on chitooligosaccharides with DP > 3.

**Figure 3:**
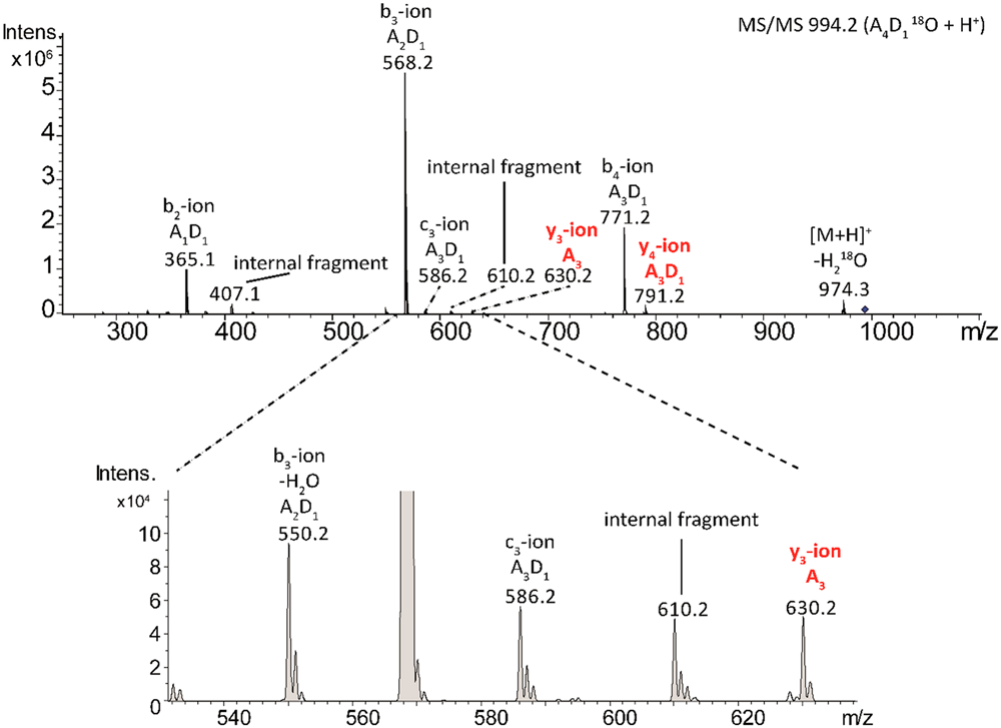
MS/MS spectrum of the mono-deacetylated product (ADAAA) from the PcCDA reaction with (GlcNAc)_5_. The reaction mixture contained mainly mono-deacetylated product and traces of di-deacetylated product. The mixture was subjected to the procedure reported in^40^. After reducing-end labelling with H_2_(^18^O), the sample was analyzed by UHPLC-ESI-MS^2^. Fragmentation spectrum of the mono-deacetylated product (A_4_D_1_^18^O): b-ions are fragments from the non-reducing end, and y-ions are fragments from reducing end with the ^18^O label.

The deacetylation pattern of the products was determined by MALDI-TOF-MS/MS sequencing^39,40^ using a preparative reaction with pentaacetylchitopentaose (A5) as substrate. A single mono-deacetylated product (ADAAA) was detected (Fig. 3). The di-deacetyated product (5% in this sample) was ADDAA. Therefore the enzyme starts deacetylating specifically the penultimate residue from the non-reducing end, and continues to the next residue towards the reducing end. No other products were detected under these experimental conditions.

### Sequence aligments and phylogenetic analysis

**Figure 4:**
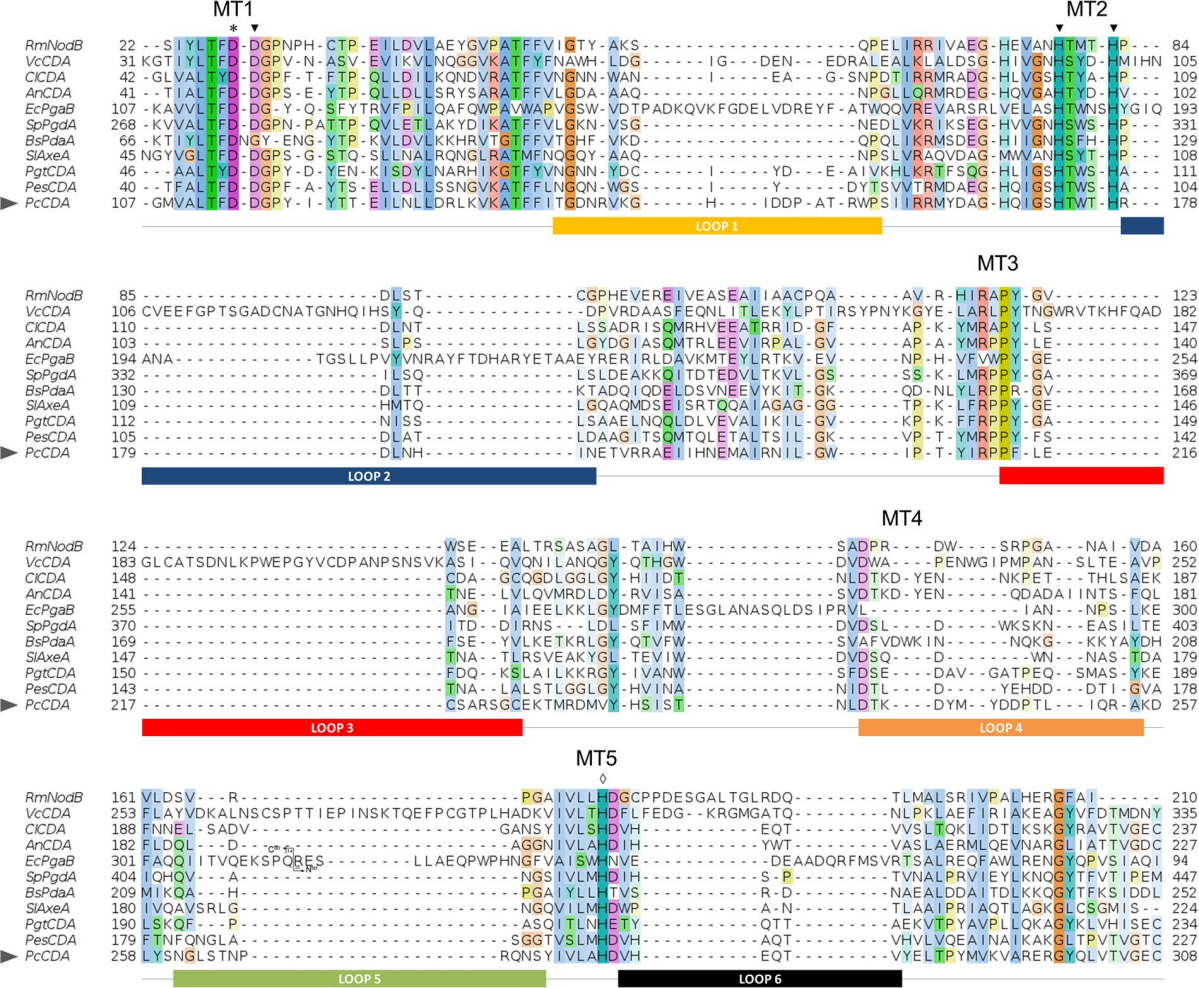
Multiple sequence alignment of chitin deacetylase (CDA) catalytic domains. Abbreviations: *Cl*CDA (*Colletotrichum lindemuthianum*), *An*CDA (*Aspergillus nidulans*), *Pes*CDA (*Pestalotiopsis* sp.), *Bs*PdaA (*Bacillus subtilis*), *Pgt*CDA (*Puccinia graminis*), *Vc*CDA (*Vibrio cholerae*), *Rm*NodB (*Rhizobium meliloti*), *Sl*AxeA (*Streptomyces lividans*), *Sp*PgdA (*Streptococcus pneumoniae*), *Ec*PgaB (*Escherichia coli*) and *Pc*CDA (*Pochonia chlamydosporia*). Loops are highlighted with colored boxes according to^28^. The arrowhead indicates the sequence of *Pc*CDA catalytic domain. Conserved catalytic motifs are labelled MT1-5. The ‘His-His-Asp’ metal binding triad (▼), catalytic base (*), and catalytic acid (¸) are highlighted.

*Pc*CDA catalytic domain sequence was added to the multiple sequence alignment of CE4 enzymes active on chitooligosaccharides guided by the structural superimposition of available X-ray structures^28^. As seen in Fig. 4, the enzyme shows conservation of the active site motifs MT1–5 characteristic of CE4 enzymes^41^. Specifically, the TFDD (MT1) motif includes the general base aspartate (first D) and the metal-binding aspartate (second D), and motif MT2 (H(S/T)xxH) contains two histidines which, together with the Asp of MT1, form the so called His-His-Asp metal-binding triad of CE4 enzymes. Since the protein here studied was refolded in the presence of Zn^2+^, the native metal of *Pc*CDA is unknown. Finally, MT5 contains the general acid histidine for catalysis. The special disposition of the catalytic residues and metal-binding triad are presented below in the structural model of *Pc*CDA.

**Figure 5:**
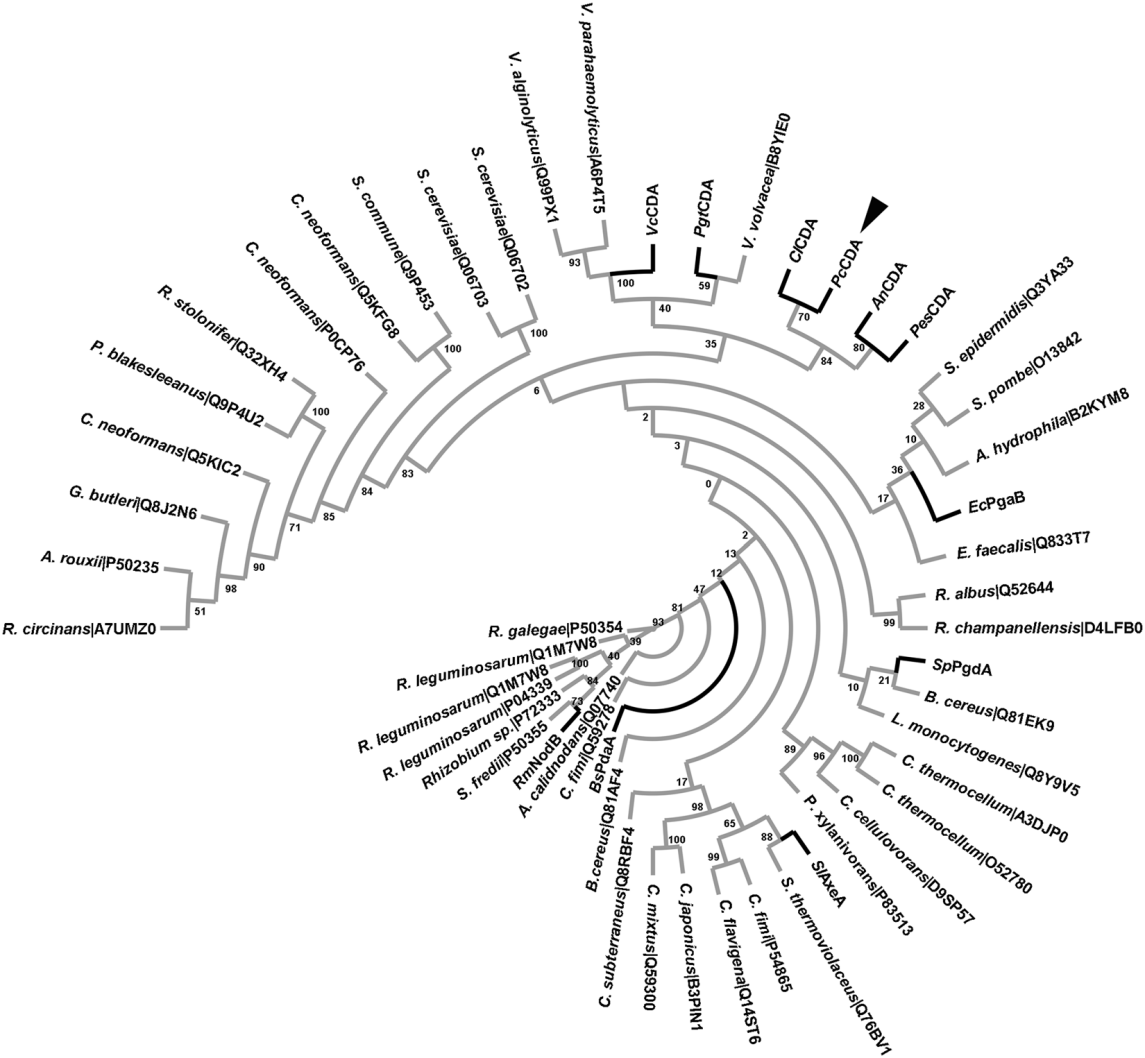
Phylogram of CE4 catalytic domains. Amino acid sequences of characterized CAZYmes from family CE4 were retrieved from Uniprot database. The enzymes reported experimentally to be active on chitooligosaccharides are highlighted using bold branches. Abbreviations: *Cl*CDA (*Colletotrichum lindemuthianum*), *Pc*CDA (*Pochonia chlamydosporia*), *An*CDA (*Aspergillus nidulans*), *Pes*CDA (*Pestalotiopsis* sp.), *Bs*PdaA (*Bacillus subtilis*), *Pgt*CDA (*Puccinia graminis*), *Vc*CDA (*Vibrio cholerae*), *Rm*NodB (*Rhizobium meliloti*), *Sl*AxeA (*Streptomyces lividans*), *Sp*PgdA (*Streptococcus pneumoniae*), and *Ec*PgaB (*Escherichia coli*).

Using the protein sequences of characterized CAZymes from family CE4, the phylogenetic relationship (Fig. 5) showed that *Colletotrichum lindemuthianum* CDA (*Cl*CDA) is *Pc*CDA closest relative, sharing 43% sequence identity. Fungal CDAs seem to be grouped and segregated from the rest of the characterized CE4 enzyme members.

### Structural model and substrate binding

**Figure 6:**
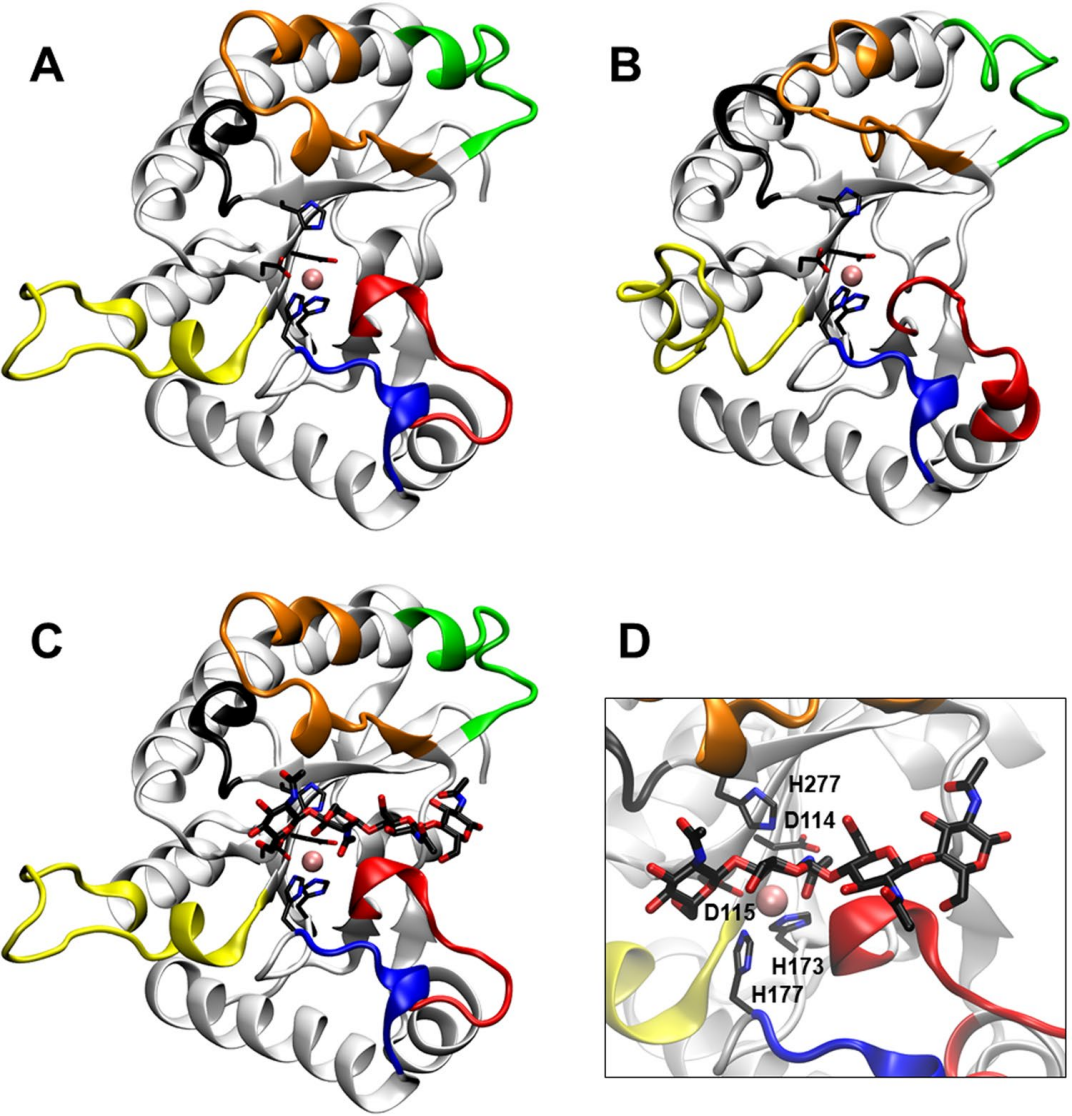
Structural models of the *Pc*CDA catalytic domain. (**A**) Model 1, using *Cl*CDA (PDB 2IW0) and *An*CDA (PDB 2Y8U) as templates. (**B**) Model 2, using *Cl*CDA (PDB 2IW0) and *Vc*CDA (PDB 4OUI) as templates. The loops are coloured as in Fig. 4 according to^28^. (**C**) Simulated docking of A4 ligand to Model 1, lowest energy binding mode which places the penultimate GlcNAc residue properly oriented for catalysis in subsite 0. (**D**) Magnification of the active site in Model 1, showing residues Asp^115^-His^173^-His^177^ (metal binding triad), Asp^114^ (general base), His^277^ (general acid), and the Zn^2+^ cation.

*C. lindemuthianum* CDA is the closest homologous protein with a solved 3D structure to the *Pc*CDA catalytic domain, followed by the *Aspergillus nidulans* CDA. Both CDAs were used to build a first structural model by homology modelling. Loop 1 (amino acids 56 to 73, Fig. 4) is longer in *Pc*CDA than in the templates and it was refined to the best empirical scoring energy, resulting in Model 1 shown in Fig. 6A. Since loop 1 has a similar length to the *Vc*CDA protein, a second model using a combination of templates (*Cl*CDA + *Vc*CDA) was built to give Model 2, which does not leave any relevant part of the *Pc*CDA sequence without templates (Fig. 6B). Both models are essentially identical along the protein structure, except for Loop 1, which appeared in two distinct conformations, extended and closed, respectively. The conformation of the loop could not be accurately determined in these calculations, being highly dependent on the template used. However, these different conformations are suggestive of intrinsic loop flexibility since no extensive interactions with core protein residues were observed in any of the models. The overall structure exhibits the canonical (β/α)_8_ fold of CE4 enzymes and has the Zn^2+^ coordination and conserved catalytic residues properly oriented in the active site (Fig. 6D).

Ligand binding was simulated by computational docking of the A4 substrate to Model 1. The preferential binding mode of the ligand placed the substrate with the non-reducing end GlcNAc residue in subsite -1 (subsites numbering as previously defined)^42^ (Fig. 6C), which is consistent with the experimentally observed first deacetylation event leading to ADAAA with the A5 substrate.

## Discussion

Few CDAs have been biochemically characterized and only four crystallographic structures have been reported, those from *Colletotrichum lindemuthianum* (first structure of a CDA)^34^, *Aspergillus nidulans*^32^, *Vibrio cholera*^28^ (and its ortholog from *V. parahaemolyticus*)^43^, and just recently, from a marine *Arthrobacter* species^44^. Efforts are directed to characterize novel CDAs elucidating the determinants of their activity, specificity and deacetylation pattern, and to use them as biocatalysts for the preparation of pure chitosan oligosaccharides with defined structures. These pure compounds rather than mixtures are necessary to assay their bioactivities and implement applications in agriculture, medicine, cosmetics, and food sciences^17,26,45^. To our knowledge, this is the first report on a chitin deacetylase from nematophagous fungi. The *P. chlamydosporia* genome encodes for three putative CDAs classified in carbohydrate esterases family 4 (CE4)^11^ based on their translated amino acid sequence. In this work, we report the protein purification, activity on different chitin oligomers, and de-*N*-acetylation pattern of the catalytic domain of *Pc*CDA protein encoded by the *Pc_2566* gene.

### PcCDA was isolated by refolding from inclusion bodies

Fungal proteins are difficult to express in bacterial hosts because they are often glycosylated proteins and/or contain many disulphide bonds^35^. Some fungal CDAs have been successfully expressed in *E. coli*. *Colletotricum limdemuthianum* and *Aspergillus nidulans* CDAs were obtained after protein refolding^46,47^ and CDAs from *Puccinia graminis* and *Pestalotiopsis* sp. overcame the difficult protein expression using protein fusions with maltose binding protein (MBP), which assisted folding and resulted in increased solubility and activity^26,35^. After codon optimization for expression in *E. coli*, we attempted, unsuccessfully, several protocols to express the isolated catalytic domain of *Pc*CDA, including different induction times and temperatures, and heat shock before induction. Aggregates or inclusion bodies were always obtained. Best results were achieved by denaturation and refolding to recover soluble and active protein from inclusion bodies. Although the protein was prone to aggregation during the purification protocol, it remained soluble after the final chromatographic step. The final yield of active recombinant protein was low and may be improved through further optimization or using alternative expression systems.

### PcCDA is active on COS and exhibits a novel deacetylation pattern

Whereas many fungal CDAs have been characterized on polymeric substrates (colloidal chitin, or soluble polymer glycol-chitin and CM-chitin), few CDAs have been analysed for substrate specificity and mode of action on low molecular weight COS, being the best characterized those from *Colletotrichum lindemuthianum*^46,48^, *Aspergillus nidulans*^31,32^, *Saccharomyces cerevisiae*^49^, and more recently from *Puccinia graminis*^35^, *Pestalotiopsis* sp.^26^, and *Podospora anserina*^36^. The closest homologue of *Pc*CDA is the *C.lindemuthianum Cl*CDA (Fig. 5) with 43% sequence identity. We show that *Pc*CDA deacetylates COS with DP > 3. For A4 and A5, the mono-deacetylated product predominates during the early stage of the reaction and the di-deacetylated product appears after longer incubation periods. A similar enzymatic behaviour was described for *Pes*CDA^26^ and *Cl*CDA^50^ but with different specificities. As opposed to *Cl*CDA, which is active on COS as short as N,N’-diacetylchitobiose (A2), *Pc*CDA does not deacetylate oligosaccharides shorter than DP4. *Pc*CDA initially deacetylates A5 at the penultimate GlcNAc residue from the non-reducing end (ADAAA), and then deacetylates the next residue towards the reducing end (ADDAA). No further deacetylated products were observed under the assayed conditions. In contrast, *Cl*CDA starts deacetylating the third residue from the non-reducing end on COS with DP ≥ 3, and continues with full deacetylation of the substrates. This indicates that subtle differences in the active site topology are responsible for the binding specificity and deacetylation pattern exhibited by closely related CDAs.

### The modelled structure of PcCDA supports substrate specificity

The recently proposed “Subsite Capping Model” suggests that the deacetylation pattern is dictated by critical loops that shape and differentially block accessible subsites in the binding cleft of CE4 enzymes^28^. Negative subsites accommodating GlcNAc units on the non-reducing end of the substrate are shaped by Loops 1, 2, and 6. *Pc*CDA has a distribution of short loops, similar to its closest homologue *Cl*CDA, except for Loop 1, which is longer but of equivalent length than that of *Vibrio cholerae* CDA (*Vc*CDA) (Fig. 4). According to our model, long Loop1 sequences partially block the accessibility of non-reducing end subsites. Accordingly, *Pc*CDA and *Vc*CDA produce, as first deacetylated products, chitosan oligomers with the same deacetylation pattern on the penultimate GlcNAc residue from the non-reducing end (ADAAA). However, *Pc*CDA continues by deacetylating a second residue while *Vc*CDA is highly specific for monodeacetylation. It may be speculated that, whereas Loop1 in *Vc*CDA is fixed by a network of H-bond interactions with Loops 2 and 6, *Pc*CDA Loop 1 might be more flexible as suggested by the structural models (Fig. 6A and B), since loop 1 establishes weaker interactions with neighbouring loops (in particular to shorter Loops 2 and 6 in *Pc*CDA as compared to *Vc*CDA) (*Supplementary Information* Figures S9 and S10). This may allow the exposure of an additional negative subsite for a second deacetylation to take place (ADDAA) at a much slower rate. The role of loop dynamics is currently under study to rationalize (and be able to engineer) the deacetylation pattern exhibited by different CDAs.

### What is the biological function of PcCDA?

One of the most studied biological functions of fungal CDAs is the protection of plant pathogenic fungi from being lysed, avoiding plant immunity responses^17,26,51^. Partial deacetylation of the exposed chitin polymer (protecting it from the action of secreted plant chitinases) or of the elicitor-active chitin oligomers (preventing binding to receptors) are proposed mechanisms to evade the plant immune response^52^, as suggested in *Colletotrichum* spp.^53^ and *Pestalotiopsis* sp.^26^. Fungal CDAs play also other physiological roles, participating in fungal nutrition, morphogenesis and development^17,21–23^, spore formation^24^, and germling adhesion^25^. CDAs have been described as virulence factors in animal pathogenic fungi. Cell wall chitin deacetylation seems essential for *Cryptococcus neoformans* virulence in lungs^54^. CDAs are considered putative virulence factors of entomopathogenic fungi since chitosan was detected in insect cuticle when infected by *Metarhizium anisopliae*^55^. In the case of *P. chlamydosporia*, the function of CDAs may be involved in nematode eggs infection rather than in a defense mechanism. Nematode eggshell contains chitin microfibrils^56^. *P. chlamydosporia* CDAs may act on the host nematode chitin because chitosan has been immunolocalised during infection of nematode eggs by the fungus^12^. Chitin deacetylation makes this polymer more elastic and soluble for fungal penetration using both hyphae and apressoria^1^ with concomitant degradation of eggshell components by extracellular enzymes^57^. CDA activity from *P. chlamydosporia* would not only have relevance in biotechnology and agriculture but also in human and animal health because this fungus also infects eggs from animal parasitic nematodes^58^.

## Conclusion

*P. chlamydosporia Pc_2566* encodes for an active chitin deacetylase (*Pc*CDA) potentially involved in nematode egg infection. The novel deacetylation pattern exhibited by the enzyme expands the repertoire of specific CDAs for biotechnological applications, where chitosan oligomers with defined pattern of acetylation are required to decipher structure-bioactivity relationships.

## Materials and Methods

### Analysis of *Pc_2566* gene sequence

*Pc_2566* gene sequence of *Pochonia chlamydosporia* strain 123, predicted *in silico* as a chitin deacetylase^11^, was retrieved from www.fungalinteractions.org (accession date 5 February 2015, Genebank assembly accession GCA_000411695.1). GeneMark and Augustus gene predictors were used for gene structure prediction. The presence of introns and their positions were verified using tBlastn searches (http://blast.ncbi.nlm.nih.gov/Blast.cgi) against RefSeq Representative Genome Database. The predicted protein-coding DNA sequence was then translated using Translate tool (http://web.expasy.org/translate). The multi-domain structure of *Pc*CDA protein sequence was analysed using the online bioinformatic tools Prosite (http://prosite.expasy.org/) and Superfamily (http://supfam.org/SUPERFAMILY/). Signal peptide (http://www.cbs.dtu.dk/services/SignalP/), transmembrane helices (http://www.cbs.dtu. dk/services/TMHMM/), N-glycosylation (http://www.cbs.dtu.dk/services/NetNGlyc/) and O-glycosylation (mucin-type) (http://www.cbs. dtu.dk/services/NetOGlyc/) site predictors were also used.

### Construction of a *Pc*CDA expression plasmid

A synthetic gene encoding for *Pochonia chlamydosporia* chitin deacetylase was designed using the predicted protein sequence and codon-optimised for *E. coli* expression (GeneOptimizer™ software, GeneArt® Gene Synthesis service, ThermoFisher)^59^. The signal peptide fragment was removed and restriction sites NdeI and SacI were added at the 5′ and 3′-ends, respectively. The gene was subcloned in the vector pET22b(+)StrepIIC between NdeI and SacI restriction sites. The construction of pET22b(+)StrepIIC was described earlier^39^. Briefly, the vector pET22b(+) (Novagen) was used as a template to include a StrepII encoding sequence downstream of the multiple cloning site using PCR via a 50-mer phosphorylated primer pair. Electrocompetent *E. coli* DH5α cells were transformed with the pET22b(+)StrepIIC plasmid containing the *Pc_2566* synthetic gene. Positive transformants were selected using LB medium supplemented with 0.1 mg mL^−1^ ampicillin and cells were grown in 10 mL of the same media and incubated overnight at 37 °C (200 rpm). The plasmid was then extracted using QIA Prep Spin MiniPrep Kit (Qiagen), and *Pc_2566* gene sequence was verified by DNA sequencing (Fig. S1). The sequence encoding the catalytic domain was amplified by PCR with specific primers containing NdeI and SacI restriction sites (Forward; 5′-TATGCATATGGTTCCGTATGGTCCG ATGATTACC-3′ and Reverse; 5′-TATCGAGCTCCAGACATTCACCAACGGTAACC-3′) using iProof High Fidelity PCR Kit (Bio-Rad). The PCR product was purified (GenElute^TM^ PCR Clean-Up Kit, Sigma-Aldrich) and digested with NdeI and SacI restriction enzymes (2.5 h, 37°). After purification (1% agarose gel electrophoresis and extraction with GenElute^TM^ Gel Extraction Kit (Sigma-Aldrich), the catalytic domain of *Pc_2566* gene sequence was ligated (T4 DNA ligase, Bio-Rad) to pre-digested pET22b(+)StrepIIC vector with the same restrictions enzymes at 16 °C overnight. *E. coli* DH5α cells were transformed with the ligation product, and positive transformants (ampicillin resistant) were verified by DNA sequencing. *E. coli* BL21(DE3) cells were transformed with the resulting pET22b-*Pc_2566*_CE4-StrepIIC plasmid (encoding the *Pc*CDA catalytic domain with C-terminal Step tag II sequence) for protein expression.

### Expression of *Pc*CDA catalytic domain in *E. coli*

*E. coli* BL21(DE3) cells containing the expression plasmid pET22b-*Pc_2566*_CE-StrepIIC were grown in LB medium supplemented with 0.1 mg mL^−1^ ampicillin at 37 °C under agitation (200 rpm). When cell density (A_600_) reached 0.8, the culture was induced with 0.02 mM IPTG and 2% sterile ethanol (by filtration) at 16 °C and the culture was maintained with agitation at 16 °C for 18 h. Cells were harvested by centrifugation (20 min, 5000 rpm) and stored at −20 °C until processing.

### Protein purification by refolding and affinity chromatography

Cell pellet was washed once with PBS buffer (50 mM K_2_HPO_4_, 300 mM NaCl, pH 8) containing 1 mM dithiotreitol (DTT) and then centrifuged (5000 rpm) for 20 min. The pellet was resuspended in 100 mL PBS buffer containing 1 mM DTT and 1 mM phenylmethylsulfonyl fluoride (PMSF) and lysed by sonication at 4 °C using a Soniprep 150 sonifier (7 min, 10 s ON/25 s OFF, 50% amplitude). Soluble and insoluble fractions were separated by centrifugation at 10000 rpm for 60 min. Both fractions were analysed by SDS-PAGE (14%) using Coomassie blue staining (Bio-Safe Comassie G-250 Stain, Bio-Rad).

The insoluble fraction obtained from ca. 4 g of cells was washed twice with 20 mL of PBS buffer (50 mM K_2_HPO_4_, 300 mM NaCl, pH 8) containing 1 mM DTT and 1% Triton X-100 and then centrifuged at 15000 rpm for 20 min. The resulting pellet was resuspended, washed twice with PBS buffer containing 1 mM DTT followed by centrifugation (15000 rpm; 20 min). The final pellet was resuspended in 15 mL of a solution of 7 M urea, 1 mM DTT in PBS buffer and incubated at 4 °C for 30 min with shaking (150 rpm). After centrifugation (14800 rpm; 35 min), the solubilised inclusion bodies were refolded by dialysis against 1 L of PBS buffer with 1 mM DTT and 1 mM ZnCl_2_ at 4 °C to remove urea (two buffer changes, 2 h each step) followed by dialysis against PBS buffer containing 1 mM ZnCl_2_ overnight. Some protein precipitated and was removed by centrifugation (10 min at 15000 rpm, 4 °C). The final supernatant was sonicated for 1 min, 0.45 µm filtered and stored at 4 °C until used.

The refolded *Pc*CDA catalytic domain was purified by affinity chromatography on an ÄKTA Protein Purification System (Amersham Biosciences) using a StrepTrap column (GE Healthcare). The protein was eluted with 2.5 mM d-Desthiobiotin in PBS buffer. The protein-containing fractions were combined, and the buffer was exchanged (PBS buffer) and the protein concentrated up to 2 mL using an Amicon Ultra-15 Centrifugal Filter, (Millipore). Protein concentration was determined with the BCA Protein Assay Kit (ThermoFisher).

### Analysis of chitin deacetylase activity of *Pc*CDA catalytic domain

Chitin deacetylase reactions were performed at final concentrations of 0.2 mM GlcNAc_5_ (A5), GlcNAc_4_ (A4) or GlcNAc_3_ (A3) substrates, 3.2 nM protein, in 50 mM K_2_HPO_4_, 300 mM NaCl, buffer at pH 8 in a total volume of 200 µL at 37 °C. At different time intervals, 10 μL aliquots were withdrawn and mixed with 90 μL of H_2_0:propanol (1:1) in HPLC vials to stop the reaction. Samples were analysed by HPLC-MS (HPLC 1200, ESI-MS 6100 series SQ, Agilent Technologies) using a XBridge BEH Amide 2.5 μm 3.0 × 100 mm Column XP, (Waters) in combination with a XBridge Amide Guard Cartridge (2PK) pre-column (2.5 µm 4.6 × 20 mm; Waters), 5 μL injection, and isocratic elution at 60 °C with acetonitrile/water 65:35 v/v, 0.1% formic acid, at a flow rate of 0.4 ml/min. MS detection monitored (SIM mode) the following [M+H]^+^ ion masses: m/z 628 (A3), 586 (A2D1), 544 (A1D2), 831 (A4), 789 (A3D1), 747 (A2D2), 1034 (A5), 992 (A4D1) and 950 (A3D2). Data was analysed with the ChemStation Software (Agilent Technologies).

### Pattern of deacetylation by *Pc*CDA catalytic domain

A preparative reaction was performed by incubating 0.3 mg of freshly prepared *Pc*CDA catalytic domain protein and 5 mg pentaacetylchitopentaose (A5) substrate in PBS buffer (50 mM K_2_HPO_4_, 300 mM NaCl, pH 8) at 37 °C, in a final volume of 1 mL. After 48 h reaction time, the sample mostly contained mono-deacetylated product and traces of di-deacetylated product (as determined by HPLC-MS as above). The mixture was analysed following the procedure reported in^40^. Briefly, the freeze-dried sample was subjected to reducing-end labelling with H_2_(^18^O) and analysed by UHPLC-ESI-MS^2^, where each labelled product was sequenced by fragmentation in the MS/MS analyser. The fragmentation spectrum of the mono-deacetylated product is shown in Fig. 3.

### Multiple sequence alignment and phylogenetic analysis

*Pc*CDA catalytic domain protein sequence was incorporated to the multiple sequence alignment of CE4 deacetylases active on chitooligosaccharides previously reported^28^ by hidden Markov model comparisons using HMMER^60^. *Pc*CDA was analysed together with *Cl*CDA (*Colletotrichum lindemuthianum*, accession O87119), *An*CDA (*Aspergillus nidulans*, accession Q5AQQ0), *Pes*CDA (*Pestalotiopsis* sp., accession A0A1L3THR9), *Bs*PdaA (*Bacillus subtilis*, accession O34928), *Pgt*CDA (*Puccinia graminis*, accession E3K3D7), *Vc*CDA (*Vibrio cholera*, accession Q9KSH6), *Rm*NodB (*Rhizobium meliloti*, accession P02963), *Sl*AxeA (*Streptomyces lividans*, accession Q54413), *Sp*PgdA (*Streptococcus pneumoniae*, accession Q8DP63) and *Ec*PgaB (*Escherichia coli*, accession P75906) CDA protein sequences. Sequence alignment is shown in Fig. 4.

Amino acid sequences of characterized family CE4 CAZYmes (www.CAZY.org) were retrieved from Uniprot database (August 2017). The phylogenetic relationships were inferred by using the Maximum Likelihood method based on the JTT matrix-based model^61^. Bootstrap analysis consisted of 500 replicates. The evolutionary analysis was conducted in MEGA7^62^, and the output dendrogram shown in Fig. 5.

### Molecular modelling of the *Pc*CDA catalytic domain and ligand docking

The three dimensional (3D) structure of *Pc*CDA catalytic domain was modelled by means of homology modelling using the HHPRED server^63^ and MODELLER software^64,65^. The X-ray structures of *Cl*CDA (PDB: 2IW0), *An*CDA (PDB: 2Y8U), and *Vc*CDA (PDB: 4OUI, in complex with A3) were selected as templates for the threading. The model was refined with a short simulated annealing protocol as implemented in MODELLER. Both 3D models, Model 1 using 2IW0 and 2Y8U as templates, and Model 2 using 2IW0 and 4OUI as templates, rendered an ensemble of conformations for Loop 1. The final structure models were assessed by means of empirical scoring energies with the DOPE score^66^.

The preferential binding modes of tetraacetylchitotetraose (A4) on to *Pc*CDA Model 1 structure were evaluated by means of virtual docking with AutoDock VINA algorithm^67^. The structure of the A4 ligand was extracted from PDB 1LZC. Both the protein and ligand structures were first parametrized: polar hydrogens were added with AutoDockTools^68^, AutoDock4.2 atom typing was used, and Gaisteger partial charges were computed for each atom with AutoDockTools. All rotatable bonds of the ligand were considered free during the docking calculations, whereas the whole protein structure was kept fixed. A grid-box of 34.5 × 33.75 × 24 Å^3^ centered at the active site was used as the search space for docking. The search for 20 different binding modes was requested with an exhaustiveness parameter set to 24. 3D structures were analyzed with the VMD visualization software^69^.

### Data Availability

All data generated or analysed during this study are included in this published article (and its Supplementary Information file).

## Electronic supplementary material

Supplementary Information
