# Supplementary material for: Expression and specificity of a chitin deacetylase from the nematophagous fungus Pochonia chlamydosporia potentially involved in pathogenicity
Source: Sci Rep. 2018 Feb 1;8:2170. doi: 10.1038/s41598-018-19902-0 (PMC5794925; doi:10.1038/s41598-018-19902-0)

Supplementary Information

Expression and specificity of a chitin deacetylase from the nematophagous fungus *Pochonia chlamydosporia* potentially involved in pathogenicity

Almudena Aranda-Martinez[1], Laia Grifoll-Romero[2], Hugo Aragunde[2], Enea Sancho-Vaello[2], Xevi Biarnés[2], Luis Vicente Lopez-Llorca[1], and Antoni Planas[2]*

[1]Laboratory of Plant Pathology, Department of Marine Sciences and Applied Biology, Multidisciplinary Institute for Environmental Studies Ramón Margalef, University of Alicante, PO box 99, 03080 Alicante, Spain. [2]Laboratory of Biochemistry, Institut Químic de Sarrià, Universitat Ramon Llull, Via Augusta 390, 08017, Barcelona, Spain.

Contents:

Figure S1. *Pc_2566* nucleotide sequence and predicted protein sequence.

Figure S2. Augustus and GeneMark output for *pc_256*6 gene prediction.

Figure S3. *Pochonia chlamydosporia* (*pc_2566*) and *Metarhizium acridum* (NW_006916702.1) chitin deacetylase (CDA) genomic sequence alignment.

Figure S4. pET22b-*Pc_2566*_CE-StrepIIC plasmid for *Pc*CDA protein expression.

Figure S5. SDS polyacrylamide gel electrophoresis analyses of protein expression and refolding.

Figure S6. HPLC-MS monitoring of deacetylase activity by *Pc*CDA catalytic domain on $GlcNAc_4$.

Figure S7. HPLC-MS monitoring of $GlcNAc_3$ incubated with the *Pc*CDA catalytic domain.

Figure S8. Sequence alignment of *Pc*CDA from *P. chlamydosporia* (catalytic domain) and *Cl*CDA from *C. lindemuthianum.*

Figure S9. Structural models of *Pc*CDA catalytic domain in complex with substrates.

Figure S10. Model structures of *Pc*CDA. Superposition of Models 1 and 2.

Figure S11. Original SDS-PAGE gels used in Figure S5.

Figure S1. (A) Pc_2566 nucleotide sequence and predicted protein sequence. Signal peptide is shown in italics. Double underline shows the catalytic domain and single underline indicates the position of the CBM18 predicted modules. Circle and square boxes point putative O-glycosylation and N-linked glycosylation sites, respectively. Cysteine residues are highlighted with (~) symbols. The position of introns is indicated by arrows in the nucleotide sequence (see Fig. S2 for full genomic sequence) while (*) symbol indicates the stop codon. (B) *Pc*CDA protein domains. Modular structure of *Pc*CDA protein showing both CBM18 domains flanking the CE4 catalytic domain and signal peptide in the N-terminal end, and structure of expressed protein purified by affinity chromatography containing CE4 domain and Strep Tag peptide.

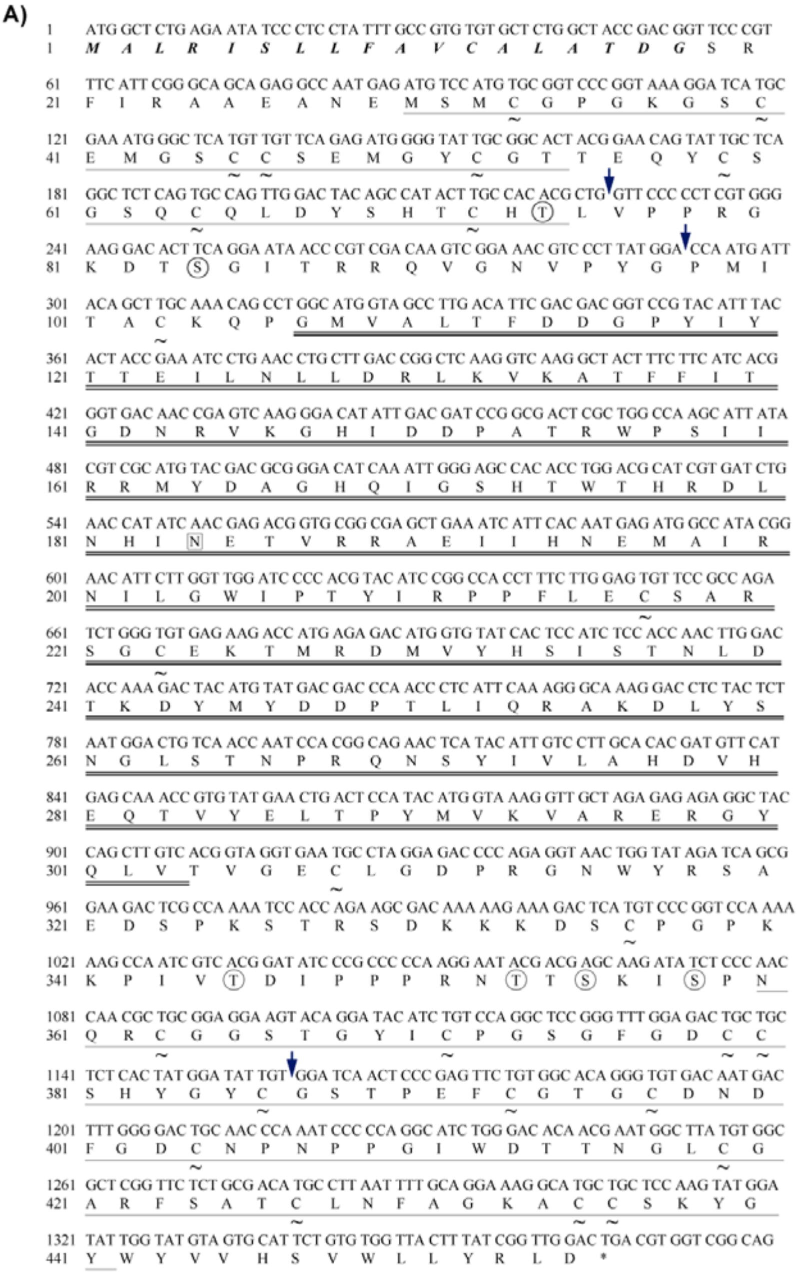

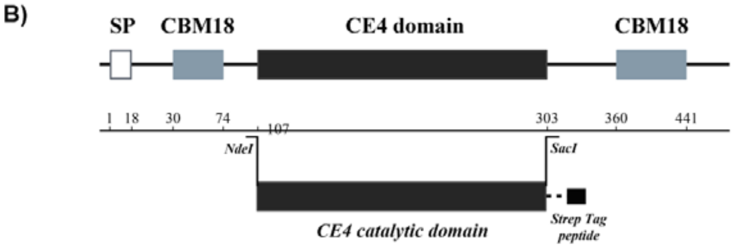

Figure S2. Augustus and GeneMark output for Pc_2566 gene prediction. (A) Augustus predicts 5 introns while (B) GeneMark only 4. Two first introns are coincident in size and position.

A. Augustus output for Pc_2566 gene prediction

unnamed-1	AUGUSTUS	gene	2596	4297	0.07	+	.	g2
unnamed-1	AUGUSTUS	transcript	2596	4297	0.07	+	.	g2.t1
unnamed-1	AUGUSTUS	start_codon	2596	2598	.	+	0	transcript_id "g2.t1"; gene_id "g2";
unnamed-1	AUGUSTUS	initial	2596	2819	0.26	+	0	transcript_id "g2.t1"; gene_id "g2";
unnamed-1	AUGUSTUS	internal	2910	2974	0.5	+	1	transcript_id "g2.t1"; gene_id "g2";
unnamed-1	AUGUSTUS	internal	3031	3653	0.64	+	2	transcript_id "g2.t1"; gene_id "g2";
unnamed-1	AUGUSTUS	internal	3859	3898	0.45	+	0	transcript_id "g2.t1"; gene_id "g2";
unnamed-1	AUGUSTUS	internal	3977	4144	0.36	+	2	transcript_id "g2.t1"; gene_id "g2";
unnamed-1	AUGUSTUS	terminal	4200	4297	0.4	+	2	transcript_id "g2.t1"; gene_id "g2";
unnamed-1	AUGUSTUS	intron	2820	2909	0.48	+	.	transcript_id "g2.t1"; gene_id "g2";
unnamed-1	AUGUSTUS	intron	2975	3030	0.46	+	.	transcript_id "g2.t1"; gene_id "g2";
unnamed-1	AUGUSTUS	intron	3654	3858	0.46	+	.	transcript_id "g2.t1"; gene_id "g2";
unnamed-1	AUGUSTUS	intron	3899	3976	0.53	+	.	transcript_id "g2.t1"; gene_id "g2";
unnamed-1	AUGUSTUS	intron	4145	4199	0.42	+	.	transcript_id "g2.t1"; gene_id "g2";
unnamed-1	AUGUSTUS	CDS	2596	2819	0.26	+	0	transcript_id "g2.t1"; gene_id "g2";
unnamed-1	AUGUSTUS	CDS	2910	2974	0.5	+	1	transcript_id "g2.t1"; gene_id "g2";
unnamed-1	AUGUSTUS	CDS	3031	3653	0.64	+	2	transcript_id "g2.t1"; gene_id "g2";
unnamed-1	AUGUSTUS	CDS	3859	3898	0.45	+	0	transcript_id "g2.t1"; gene_id "g2";
unnamed-1	AUGUSTUS	CDS	3977	4144	0.36	+	2	transcript_id "g2.t1"; gene_id "g2";
unnamed-1	AUGUSTUS	CDS	4200	4294	0.4	+	2	transcript_id "g2.t1"; gene_id "g2";
unnamed-1	AUGUSTUS	stop_codon	4295	4297	.	+	0	transcript_id "g2.t1"; gene_id "g2";

B. GeneMark output for Pc_2566 gene prediction

Gene #	Exon #	Strand	Exon Type	Exon Range		Exon Length	Start/End Frame
3	1	+	Initial	2596	2819	224	1 2 - -
3	2	+	Internal	2910	2974	65	3 1 - -
3	3	+	Internal	3031	3653	623	2 3 - -
3	4	+	Internal	3700	4144	445	1 1 - -
3	5	+	Terminal	4200	4297	98	2 3 - -

Figure S3. *Pochonia chlamydosporia* (Pc_2566) and *Metarhizium acridum* (NW_006916702.1) chitin deacetylase (CDA) genomic sequence alignment. Introns of *M. acridum* sequence are marked in yellow. First two introns (red bars) predicted by both Augustus and GeneMark are coincident. Third intron (green bars) from *P. chlamydosporia* was predicted taking into account the position of *M. acridum* third intron.

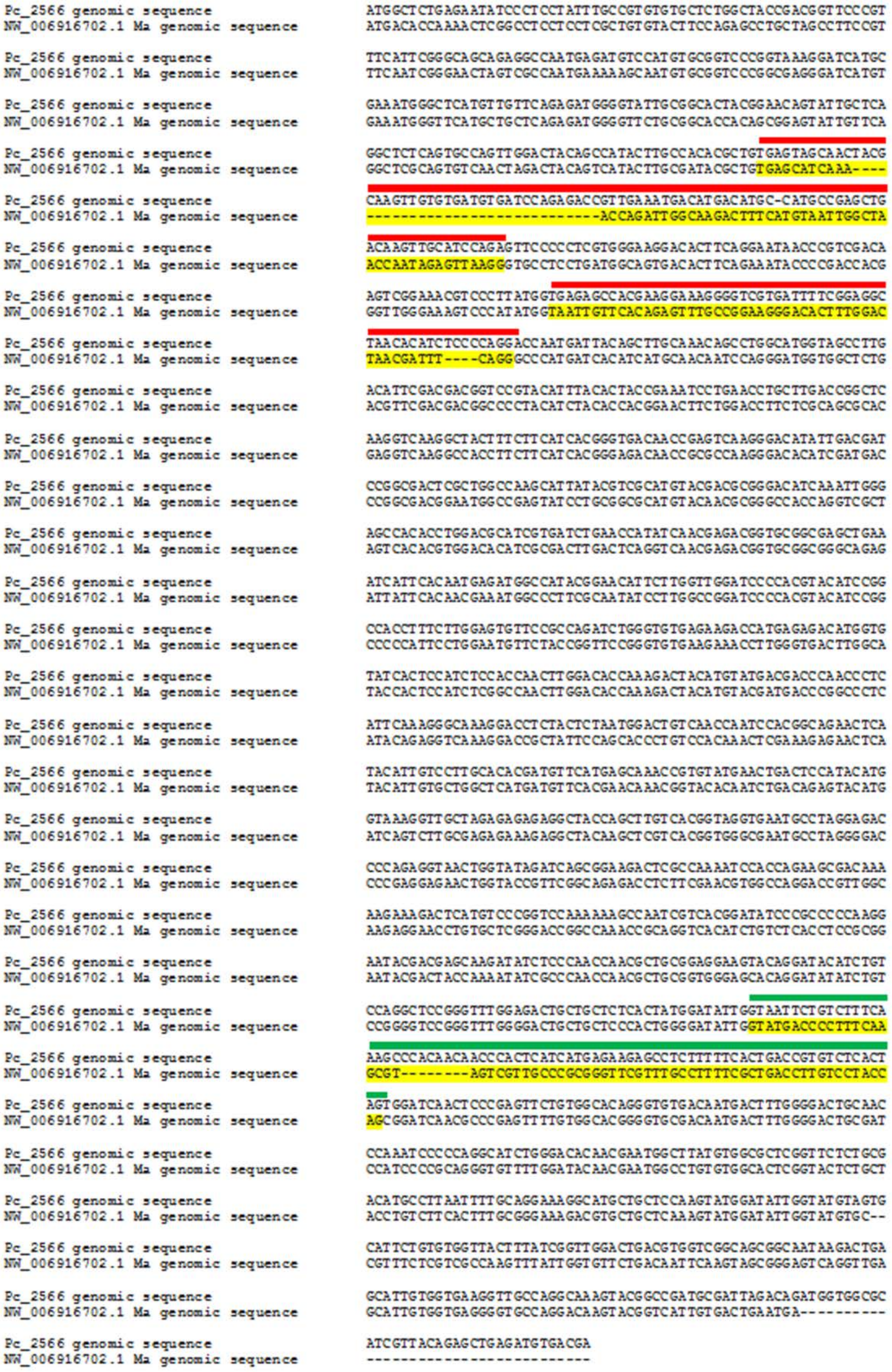

Figure S4. pET22b-*Pc_2566*_CE-StrepIIC plasmid for *Pc*CDA protein expression. The plasmid contains the *Pc*CDA CE4 domain and the Strep tag peptide coding sequences followed by a stop codon before the His tag codifying peptide of the original pET22b(+) vector.

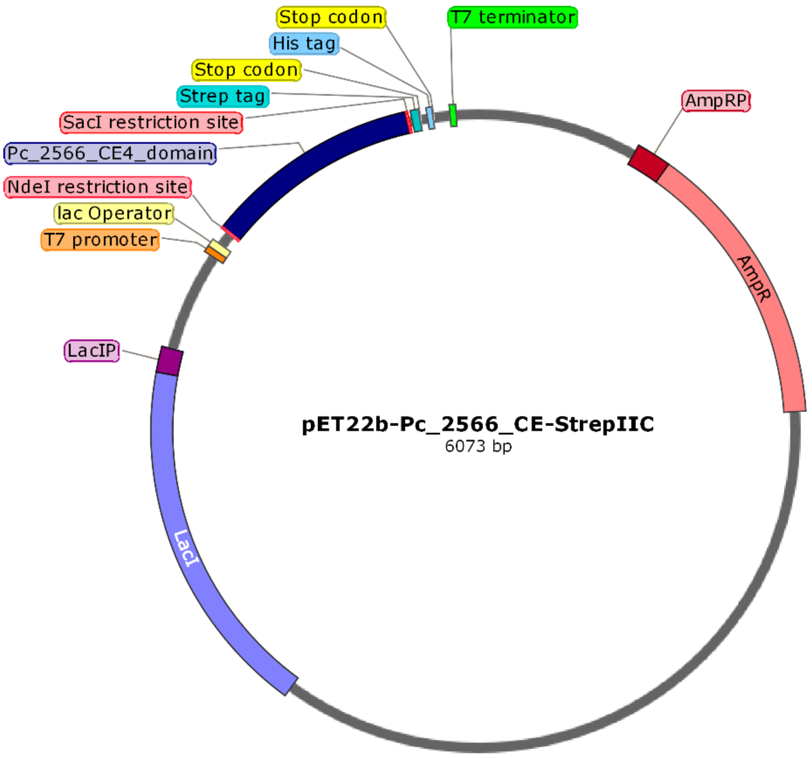

Figure S5. SDS polyacrylamide gel electrophoresis analyses of protein expression and refolding. A) Proteins from induced *E. coli* BL21 strain transformed with pET22b-Pc_2566_CE4. Lane S: supernatant (soluble fraction), Lane P: insoluble fraction (pellet) after cell disruption. B) Protein refolding and purification. Lane 1, culture supernatant (soluble fraction). Lanes 2 and 3, pellet after Triton X100 treatment. Lanes 4 and 5, samples after two washes to remove Triton X100. Lane 6, supernatant after urea treatment for protein solubilisation. Lane 7, pellet obtained after urea treatment. Lane 8, sample after protein refolding by dialysis. Arrowheads indicate expected size of *Pc*CDA catalytic domain. Original gels in Fig. S11.

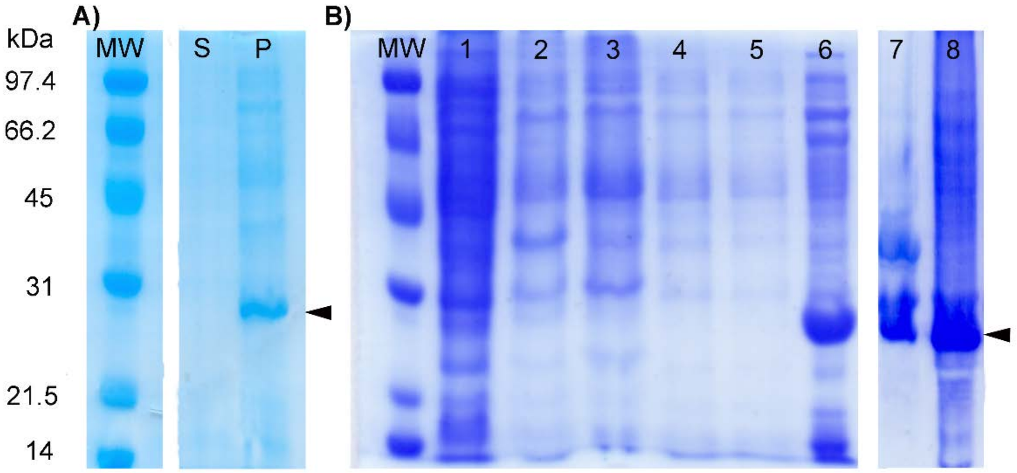

Figure S6. HPLC-MS monitoring of deacetylase activity by *Pc*CDA catalytic domain on GlcNAc$_4$. Chromatograms show the presence of the starting product (A4) and the formation of mono-deacetylated (A3D1) and di-deacetylated (A2D2) products at different reaction times: (A) 1 h, (B) 16 h, (C) 24 h and (D) 100 h. Reaction conditions: 0.2 mM substrate, 3.2 nM enzyme, 50 mM K_2HPO_4, 300 mM NaCl, pH 8.0, 37ºC.

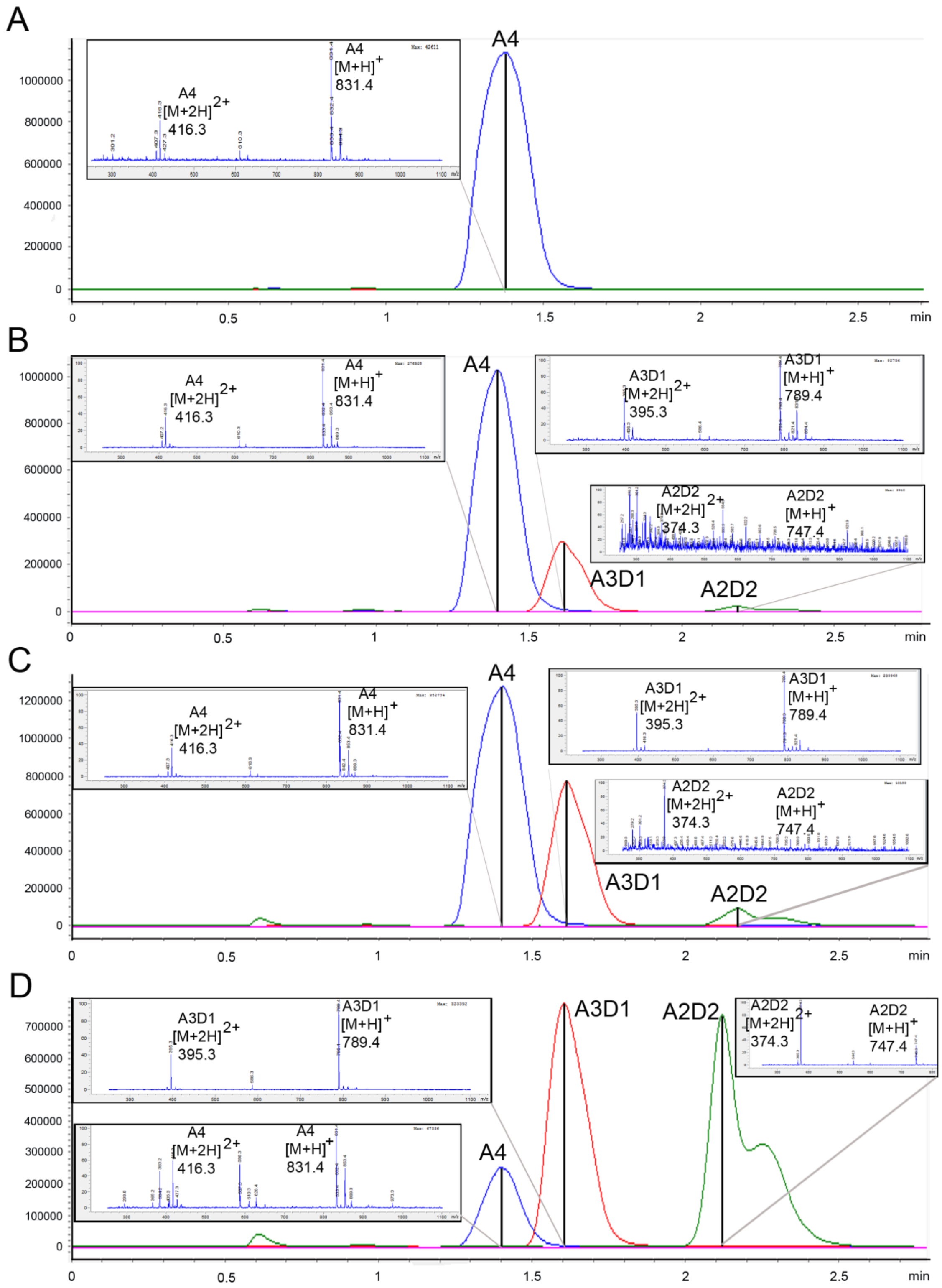

Figure S7. HPLC-MS monitoring of GlcNAc$_3$ incubated with the *Pc*CDA catalytic domain. Chromatograms show the presence of the starting product (A3) and the absence of reaction products with time: (A) 10 min, (B) 5 h, (C) 24 h and (D) 100 h. Reaction conditions: 0.2 mM substrate, 3.2 nM enzyme, 50 mM K$_2$HPO$_4$, 300 mM NaCl, pH 8.0, 37ºC.

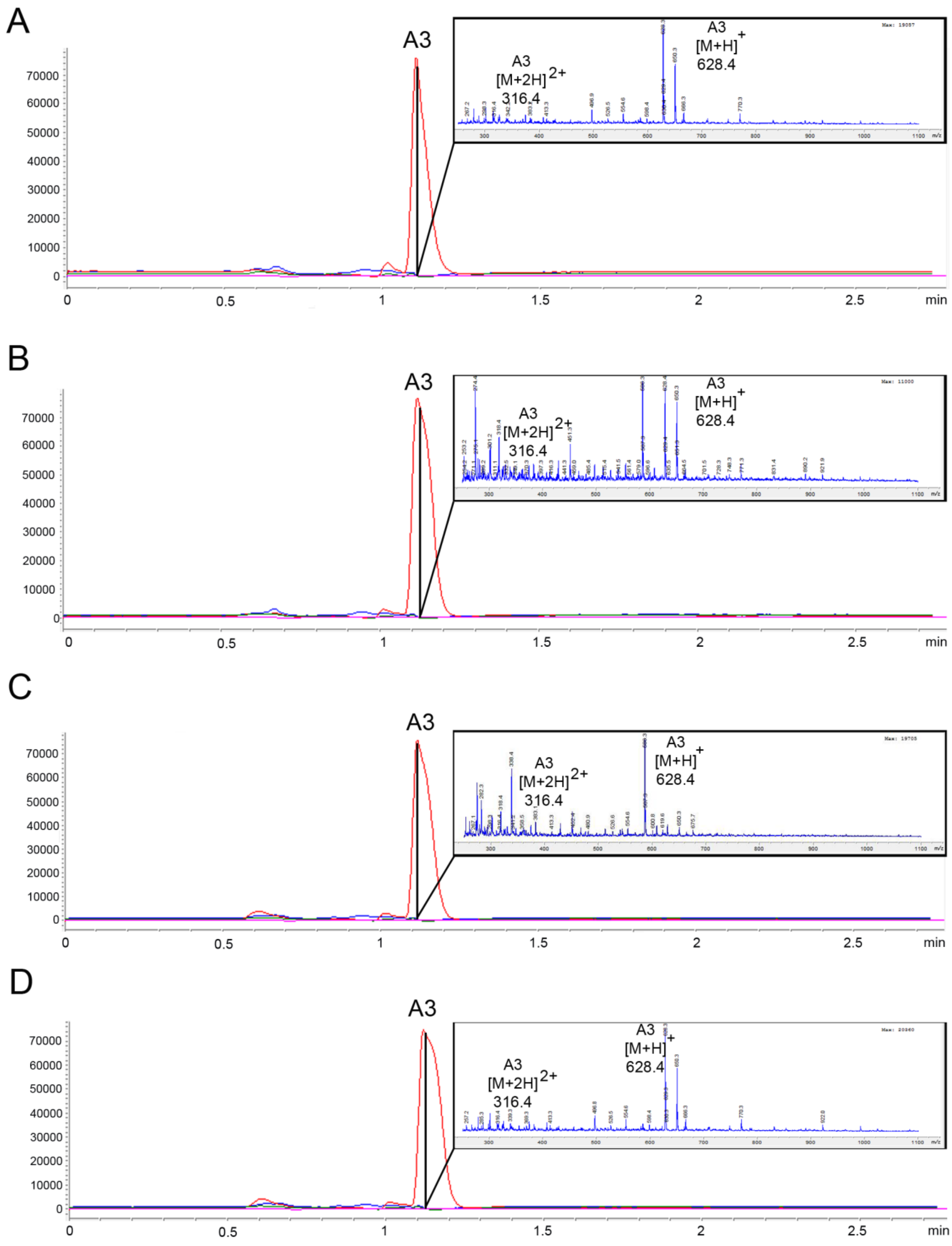

Figure S8. Sequence alignment of *Pc*CDA from *P. chlamydosporia* (catalytic domain) and *Cl*CDA from *C. lindemuthianum.*

```
Pc_2566 CDA   MALRISLLFAVCALATDGSRFIRAAEANEMSMCGPGKGSCEMGSCCSEMGYCGTTEQYCS
ClCDA         ------------------------------------------------------------

Pc_2566 CDA   GSQCQLDYSH---TCHTLVPPRGKDTSGITRRQVGNVPYGPMITACKQPGMVALTFDDGP
ClCDA         -----MHFSTLFGAAATAALAGSTNASPLARRQ---VPVGTPILQCTQPGLVALTYDDGP

Pc_2566 CDA   YIYTTEILNLLDRLKVKATFFITGDNRVKGHIDDPATRWPSIIRRMYDAGHQIGSHTWTH
ClCDA         FTFTPQLLDILKQNDVRATFFVNGNNW--ANIEAGSN--PDTIRRMRADGHLVGSHTYAH
                                    Loop 1

Pc_2566 CDA   RDLNHINETVRRAEIIHNEMAIRNILGWIPTYIRPPFLECSARSGCEKTMRDMVYHSIST
ClCDA         PDLNTLSSADRISQMRQLEEATRRIDGFAPKYMRAPYLSCDA--GCQGDLGGLGYHIIDT
               Loop 2                                  Loop 3

Pc_2566 CDA   NLDTKDYMYDDPTLIQRAKDLYSNGLSTNPRQNSYIVLAHDVHEQTVYELTPYMVKVARE
ClCDA         NLDTKDYENNKPETTHLSAEKFNNELSADVGANSYIVLSHDVHEQTVVSLTQKLIDTLKS
                    Loop 4             Loop 5          Loop 6

Pc_2566 CDA   RGYQLVTVGECLGDPRGNWYRSAEDSPKSTRSDKKKDSCPGPKKPIVTDIPPPRNTTSKI
ClCDA         KGYRAVTVGECLGDAPENWYKAHHHHHH--------------------------------

Pc_2566 CDA   SPNQRCGGSTGYICPGSGFGDCCSHYGYCGSTPEFCGTGCDNDFGDCNPNPPGIWDTTNG
ClCDA         ------------------------------------------------------------

Pc_2566 CDA   LCGARFSATCLNFAGKACCSKYGYWYVVHSVWLLYRLD
ClCDA         --------------------------------------
```

Figure S9. Structural models of *Pc*CDA catalytic domain in complex with substrates. A) Model 2 with superimposed A3 ligand from template 4OUI (VcCDA·A3 complex). B) Simulated docking of A4 ligand to Model 1. In both, A and B, the ligand binding mode places the penultimate GlcNAc residue properly oriented for catalysis in subsite 0. C) Simulated docking of A4 ligand to Model 1 in a second binding mode, which places the reducing end GlcNAc residue in subsite -2. This binding mode was only possible in Model 1 with an extended Loop 1 conformation, but not in Model 2 with a closed Loop 1 conformation. Loops are coloured as in Figures 4 and 6.

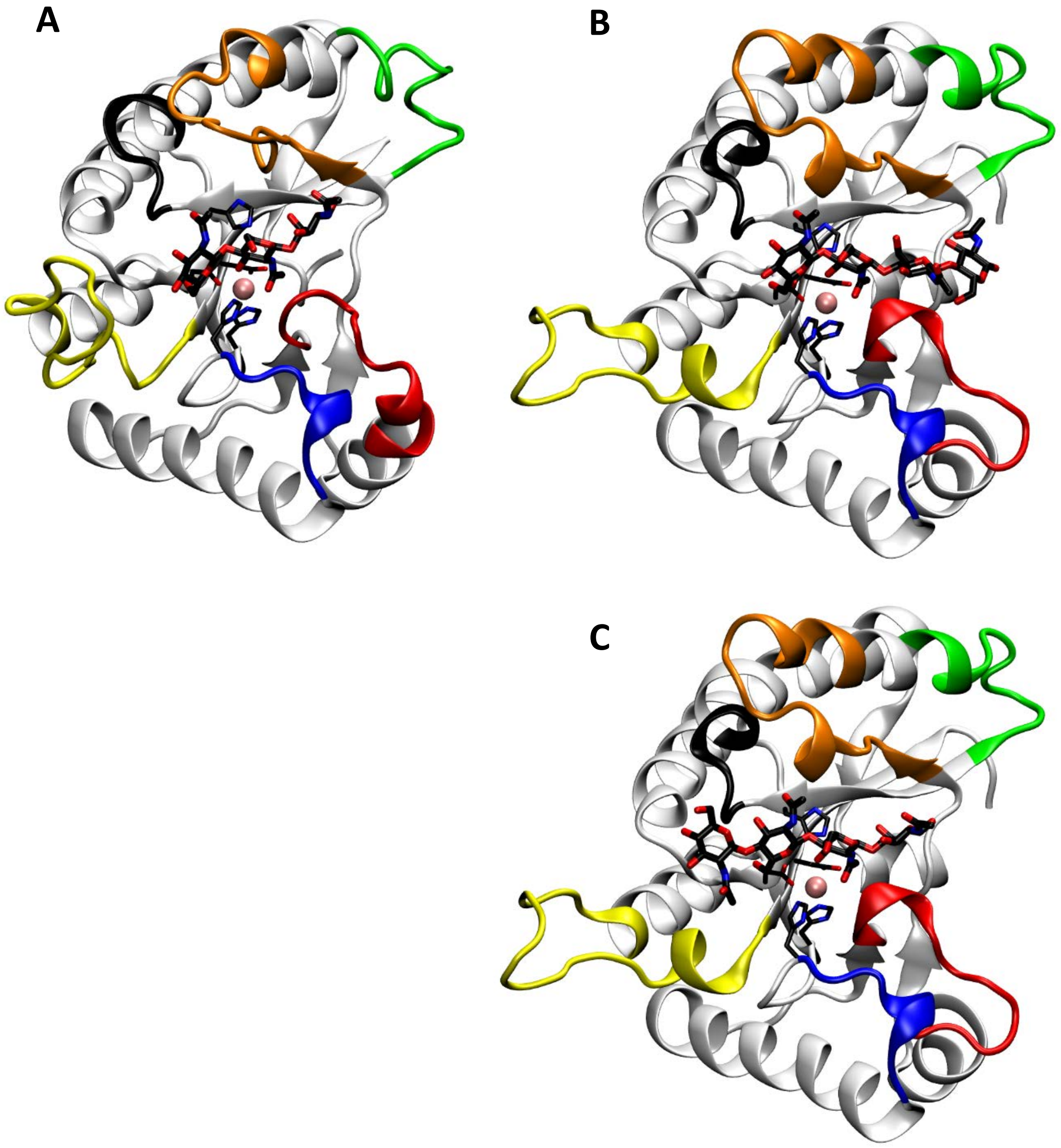

Figure S10. Superposition of Models 1 and 2 (model structures A and B in Figure S9). Both models are essentially identical along the protein structure, except for Loop 1, which appears as an ensemble of conformations in both models. Shown here in yellow, the extended (Model 1) and closed (model 2) Loop 1 conformations for the lowest energy models.

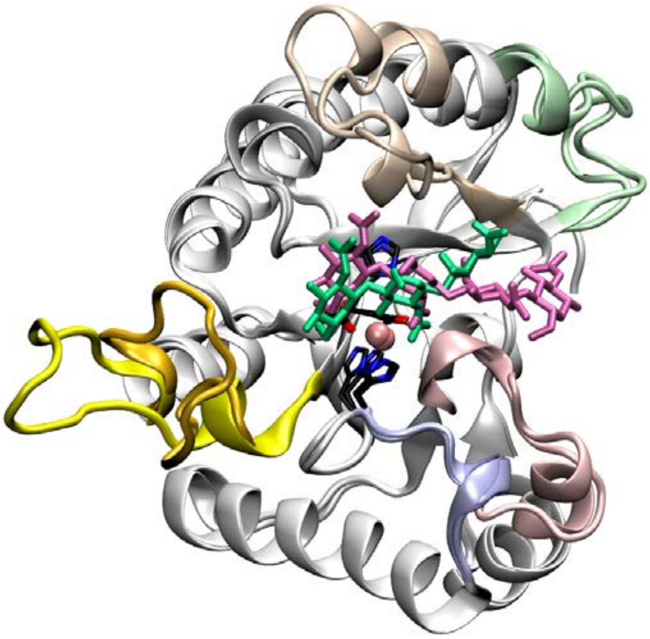

Figure S11. Original SDS-PAGE gels used in Figure S5.

A) Original gel in Figure S5.A (labelled lanes)

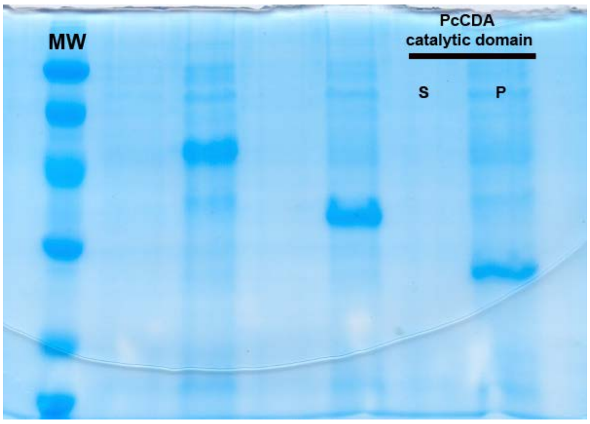

B) Original gels in Figure S5.B (labelled lanes)

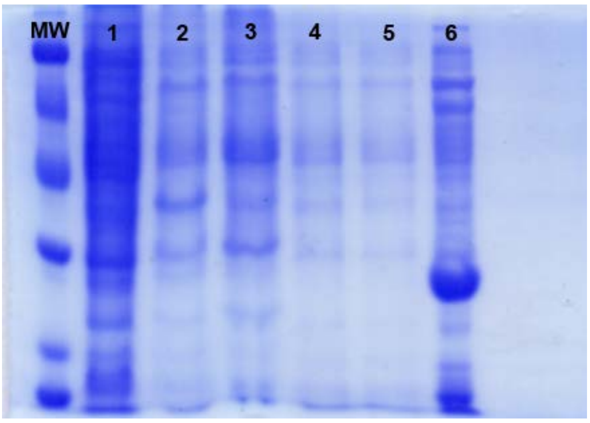

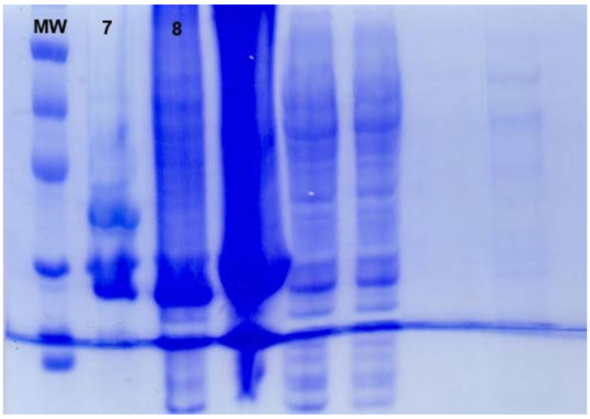

Supplement: Supplementary file 1 — Supplementary Information [file 41598_2018_19902_MOESM1_ESM.pdf]
